# Aging aggravates cognitive dysfunction in spontaneously hypertensive rats by inducing cerebral microvascular endothelial dysfunction

**DOI:** 10.1371/journal.pone.0316383

**Published:** 2025-03-13

**Authors:** Mei Wu, Dandan Li, Feng Qiu, Huifang Nie, Rui Fang, Ziyan Zhong, Hui Yang, Xiaoyuan Lin, Xiangyuan Wang, Hongbo Wen, Lijun Gong, Pan Meng

**Affiliations:** 1 Hunan University of Chinese Medicine, Changsha, Hunan, China; 2 Hunan Academy of Chinese Medicine, Changsha, Hunan, China; 3 First Affiliated Hospital, Hunan University of Chinese Medicine, Changsha, Hunan, China; 4 Yiyang Central Hospital, Yiyang, Hunan, China; Universidade Federal do Rio de Janeiro, BRAZIL

## Abstract

Hypertension in the elderly can seriously lead to cerebral microvascular damage and promote the development of vascular cognitive impairment. While endothelial function is crucial in cerebral microvascular protection, it is unclear whether aging exacerbates hypertension-induced cognitive dysfunction through endothelial dysfunction. In this study, we injected D-galactose (D-gal) into 24 spontaneous hypertension rats (SHR) and 24 Wistar-Kyoto rats (WKY) for 12 weeks to induce aging. Firstly, the results of behavioral experiments showed that compared with WKY and SHRs injected with D-gal for 0 week, SHRs injected with D-gal for 12 weeks had more severe cognitive dysfunction and memory impairment. Subsequently, the pathological results showed that the pathological changes of brain microvessels and their structural and functional damage were more significant. After that, the results of molecular experiments showed enormous changes in endothelial damage indicators (nitric oxide (NO), endothelin (ET-1), platelet endothelial cell adhesion molecule-1(CD31) and endothelial tight junction protein), aggravation of blood-brain barrier (BBB) damage, microglial activation and upregulation of pro-inflammatory cytokines. Ultimately, the combination treatment of nimodipine and butylphthalide in WKY and SHRs injected with D-gal for 12 weeks showed that the two drugs could hugely improve the cognitive dysfunction in SHRs. In summary, we elaborated that aging exacerbates cognitive dysfunction in SHRs, which may be due to cerebral microvascular endothelial dysfunction, and even BBB damage and neuroinflammation, while the combination of nimodipine and butylphthalide can improve cognitive dysfunction in SHRs, providing a theoretical basis for the treatment of aging and hypertension-related diseases.

## 1. Introduction

Hypertension has harmful effects on the brain and cerebral circulation. As the world’s population ages, hypertension-related morbidity and mortality among older adults are on the rise, with more than half of the elderly population suffering from hypertension [1]. Strong epidemiological and experimental evidence suggests that both aging and hypertension can contrubute to impaired function and structure of cerebral microvessels, deformation of blood vessels, and reduced cerebral blood flow (CBF), thereby inducing vascular cognitive impairment [2,3]. That is the second most common cause of cognitive impairment after Alzheimer’s disease. Although available human data suggest a synergistic effect between advanced age and hypertension, there are no studies exploring specific age-related mechanisms by which aging increases the vulnerability of the cerebral microvascular system to hypertension, inducing cognitive dysfunction.

Cerebral microvasculature is essential for cerebral autoregulation, but due to its fragile structure, it can cause microvascular damage in the face of changes in blood pressure and age [4]. The first cells to respond to external stimuli are endothelial cells (ECs), which normally regulate vascular tone and local blood flow by synthesizing relaxation factors such as nitric oxide (NO), endothelin (ET-1). Abnormal NO production in vascular ECs may promote endothelial dysfunction, shifting endothelial function from vasoprotective to vasoconstrictive and prothrombotic functions, ultimately resulting in hypertension and further cardiovascular complications[5,6]. This was confirmed in a series of subsequent studies. Numerous clinical studies have found that endothelial dysfunction leads to a decrease in the bioavailability of NO in patients with essential hypertension[7,8]. Another study showed similar results, with serum phosphorus levels in hypertensive patients associated with endothelial dysfunction[9]. It has also been found that NO and ET-1 change with age[10,11]. Although endothelial dysfunction has allimportant research implications in both aging and hypertension, it is unclear whether aging exacerbates hypertension-induced cognitive dysfunction by affecting endothelial function. However, nimodipine [12], which is commonly used in clinical practice for the treatment of hypertension, and butylphthalide [13] for the treatment of intracerebral hemorrhage, whether the combination of the two can improve the cognitive dysfunction in SHRs needs to be further confirmed.

This study aims to directly test the hypothesis that aging exacerbates cognitive dysfunction caused by hypertension, which may be due to aging-induced cerebral microvascular endothelial dysfunction, blood-brain barrier (BBB) disruption, and neuroinflammation. To confirm our hypothesis, we induced senescence by long-term injection of D-galactose (D-gal) in Wistar-Kyoto rats (WKY) and spontaneous hypertension rats (SHR), and assessed aging markers and systolic blood pressure, diastolic blood pressure levels, cognitive function, cerebral microvascular injury, BBB function, and neuroinflammatory markers at 0, 12 weeks of injection with D-gal, and finally treated WKY and SHRs injected with D-gal 0, 12 weeks with a combination of nimodipine and butylphthalide, and to test their cognitive function.

## 2. Methods

### 2.1. Animal model and treatment

Specific pathogen-free (SPF) 24 WKY and 24 SHRs were purchased from Beijing Vital River Laboratory Animal Technology Co., Ltd, and were raised in the Laboratory Animal Center of Hunan University of Chinese Medicine with a 12-hour light/dark cycle at 23 ± 2°C, housed 3 per cage with free access to water and food. All rats were approved by the Experimental Animal Ethics Committee of Hunan University of Chinese Medicine (Cat.: LLBH-202011090001). And the experiments were complied with all relevant codes of ethics and reported in accordance with the ARRIVE guidelines.

In this experiment, D-gal was injected subcutaneously into WKY and SHRs at 0 week (3 months old) and 12 weeks (6 months old), respectively, to induce senescence. Meanwhile, an equal amount of Normal saline (NS) was injected subcutaneously for 12 weeks as a control, followed by treatment with nimodipine and butylphthalide. After the behavioral experiment, the anesthetic material is taken and the follow-up test is carried out. This study employs intraperitoneal injection of sodium pentobarbital 1% (0.1mL/20g) for anesthesia and/or analgesia. Efforts to alleviate pain and suffering include providing appropriate analgesia and anesthesia for the duration of the experiment, as well as ensuring adequate housing conditions for the animals. After anesthetizing the animal, the abdominal aorta separates and blood is quickly drawn out, and the animal dies, followed by follow-up experimental testing.

### 2.2. Drug deliver

The dose of D-gal is 150mg/kg/day (WXBD8369V, Sigma-Aldrich). The NS dose is 150mg/kg/d (H20045210, Kelun Pharmaceutical Co., Ltd.). Nimodipine was dosed at 21.6 mg/kg (H14022821, Yabao Pharmaceutical Co., Ltd.). Butylphthalide was dosed at 54 mg/kg (H20050299, Enmepro Pharmaceutical Co., Ltd.). Sodium pentobarbital was dosed at 30mg/kg (57-33-0, Beijing Jiehui Biotechnology Co., Ltd.)

### 2.3. Experiment design

This study was divided into the following two parts.

Experiment 1: The rats were randomly divided into the following 8 group (N = 6/group):(1) WKY+NS 0w (WKY with saline injection for 0 week); (2) WKY+NS 12w (WKY with saline injection for 12 weeks); (3) WKY + D-gal 0w (WKY with D-gal injection for 0 week); (4) WKY + D-gal 12w (WKY with D-gal injection for 12 weeks); (5) SHR+NS 0w (SHR with saline injection for 0 week); (6) SHR+NS 12w (SHR with saline injection for 12 weeks); (7)SHR + D-gal 0w (SHR with D-gal injection for 0 week); (8)SHR + D-gal 12w (SHR with D-gal injection for 12 weeks).

Experiment 2: The rats were randomly divided into the following 4 groups (N = 6/group):(1) WKY + D-gal 12w (WKY with D-gal injection for 12 weeks); (2) WKY + D-gal 12w + NT + B (WKY with D-gal injection for 12 weeks, combined with the treatment of Nimodipine and butylphthalide); (3) SHR + D-gal 12w (SHR with D-gal injection for 12 weeks); (4) SHR + D-gal 12w + NT + B (SHR with D-gal injection for 12 weeks, combined with the treatment of Nimodipine and butylphthalide).

### 2.4. Tail artery pressure monitoring

The Systolic and diastolic blood pressure of rats were measured by intelligent non-invasive sphygmomanometer weekly (BP2010; Softron Beijing Biotechnology Co., Ltd.). The time of blood pressure measurement was fixed (8:00-14:00). The blood pressure measurement was repeated for 5 times within 5 minutes at an interval of 1 min. The highest and lowest outlier values were eliminated, and the average of the other 3 values was taken as the blood pressure measurement value.

### 2.5. Morris water maze (MWM)

MWM apparatus consisted of a black round stainless steel pool (depth 60 cm, diameter 150 cm), and a camera was located above the center of the maze to record the movement of the rats and send the data to data analysis software. The maze was divided into four quadrants artificially (Ⅰ、Ⅱ、Ⅲ、Ⅳ), a platform was fixed in the Ⅱ quadrant, tap water was poured into the pool, the water level was 1-2 cm higher than the platform, and the water temperature was controlled at 22-24°C. From day 1-4, the rats were allowed to perform learning training in four different quadrants for 60s. On the 5th day, the rats were put into the water in the same locations and had to swim freely for 120 s to find the platform. The time of the rat took to reach the platform was recorded (i.e., the escape latency). On the 6th day, the platform was removed, and the rats were allowed to swim freely for 120s. The latency in the target quadrant, the swimming time in the target quadrant and total swimming distance were recorded.

### 2.6. Novel Object Recognition (NOR)Test

The rats are allowed to acclimatize twice a day in an empty box (100 ×  100 ×  20 cm) for 10 min each for 3 consecutive days. Expose the rat to two similar objects for 10 min. One of the objects is then replaced with a new object of different colors and shapes. Allow the rats to explore freely for another 5 min and record the time they spend exploring each object. Recognition index used to distinguish between familiar and unfamiliar objects =  (time to explore new novel objects)/ (time to explore novel objects +  time to explore familiar objects) × 100%. Exploration time is calculated as the sum of time spent exploring familiar and novel objects.

### 2.7. Cerebral blood flow (CBF)

CBF was measured in rats using a laser speckle imaging. The rats were anesthetized and mounted on a stereotactic frame, the skull was exposed through a skin incision along the midline of the scalp and thinned with a skull drill. The laser speckle imager was placed 10 cm above the exposed skull surface. The laser cross-center was aligned with the bregma, and the whole-brain scan was performed by using LSI. Analysis was performed with moorFLPI V5.0 image.

### 2.8. Enzyme linked immunosorbent assay (ELISA)

After the animal is anesthetized with sodium pentobarbital, the abdominal aorta is separated and blood is rapidly withdrawn. Centrifuge the blood sample (5,000 rpm, 4°C, 30 min), take the supernatant and store at -80◦C.The concentrations of interleukin-6(IL-6)(E-UNEL-M0070,Elabscience), inter-cellular adhesion molecule-1 (ICAM-1)(E-EL-R2850,Elabscience), matrix metalloproteinase-9 (MMP-9)(E-EL-H1451km,Elabscience), total superoxide dismutase (T-SOD)(E-BC-K020-M,Elabscience), glutathione peroxidase (GSH-Px)(E-BC-K096-S,Elabscience), malondialdehyde (MDA)(E-BC-K025-S, Elabscience), nitric oxide (NO)(E-BC-K035-M,Elabscience), endothelin-1 (ET-1) (E-EL-H0064c, Elabscience) and CALB/SA(BES5633K, Bioesn) in serum and brain were determined by the ELISA Kit according to the manufacturer’s instructions.

### 2.9. Viscera index

After the rats were sacrificed, the brain, thymus, kidneys, and liver were thoroughly separated and their body weights was recorded. The visceral index was calculated as follows: visceral index (%) =  visceral weight (g)/ rat body weight (g) ×  100%.

### 2.10. Hematoxylin-eosin (HE) staining

The brain tissues of the rats were separated after perfusion with 0.9% normal saline and 4% cold paraformaldehyde (BL539A,Biosharp), and fixed in 4% paraformaldehyde for 24h before paraffin embedding, then 4μm sections were sliced. Frozen sections were hydrated in PBS (BL302A,Biosharp), the hydrated sections were put into hematoxylin for 1 minute, rinsed with tap water, differentiated with 1% hydrochloric acid alcohol for a few seconds, rinsed with tap water for 15 minutes, counterstained in eosin solution for a few seconds, rinsed with tap water, and observed the staining under a microscope. Ethanol gradient dehydration, 70% ethanol, 80% ethanol, 90% ethanol, absolute ethanol I, absolute ethanol II for 2 min, xylene for 5 min, neutral gum mounting.

### 2.11. Luxol fast blue (LFB) staining

Frozen sections were hydrated in PBS, and according to the instructions of the Myelin Dye Kit (G1030, Servicebio), the myelin stain solution was pre-warmed in a 65°C oven for 1 h in advance, and the hydrated sections were placed in the stain solution at 65°C for 4 h, differentiated with solution B for 5 s and solution C for 10 s, and the staining was observed under the microscope. Ethanol gradient dehydration, 70% ethanol, 80% ethanol, 90% ethanol, absolute ethanol I, absolute ethanol II for 2 min, xylene for 5 min, neutral gum mounting.

### 2.12. Immunohistochemistry

The 4μm paraffin sections were dehydrated, and the antigenic repair was performed by boiling heat repair method. The sections were sealed with normal goat serum blocking solution, and the primary antibody ZO-1 (1:3000, 402200, Invitrogen), Claudin-5 (1:3000, 352500, Invitrogen), Occludin(1:3000, ab216327, Abcam), CD31 (1:800, MA1-81051, Invitrogen) were added and placed at 4°C overnight. Then, the secondary antibody was added and incubated with biotin. Eventually, the sections are colored with DAB for ten minutes, and re-stained with hematoxylin.

### 2.13. Immunofluorescence

After the cerebral paraffin sections were deparaffinized and dehydrated, high-temperature antigen was used for retrieval and non-specific binding was blocked with 5% bovine serum albumin. Antibodies neuronal nuclear antigen (NeuN) (1:200, 26975-1-AP, Proteintech), aquaporin-4 (AQP4) (1:300, 16473-1-AP, Proteintech), glial fibrillary acidic protein (GFAP)(1:1000, GB12090, Servicebio), ionized calcium-binding adaptor molecule 1 (Iba-1) (1:2000, GB12105, Servicebio) were instilled at 4°C and incubated overnight. After primary antibody incubation, repeat PBS washes 3 times and incubate with CY3-labeled goat anti-rabbit and anti-mouse secondary antibodies for 60 min in the dark. Finally, images are observed and collected under a fluorescence microscope using an antifluorescence quenching sealant containing DAPI.

### 2.14. Western blotting(WB)

Total protein was extracted using RIPA lysate (C5029, Bioss) containing a cocktail of protease and phosphatase inhibitors. Protein concentrations were determined by BCA(E-BC-K318-M, Elabscience) assay. Proteins were separated by SDS-PAGE and transferred to polyvinylidene fluoride (PVDF) membranes. The PVDF membranes was blocked in 5% skim milk powder in TBST for 2 h at room temperature, followed by incubating with primary antibody in 5% BSA/TBST at 4 °C overnight. The primary antibodies used in this study include ZO-1 (1:3000, 402200, Invitrogen), Occludin(1:3000, ab216327, Abcam) and β-actin(1:6000, bs-10966R, Bioss).After 3 times washes with TBST, the membranes were incubated with the following secondary antibodies: Goat Anti-Mouse IgG antibody (H +  L), peroxidase (1:10000, E-AB-1001, Elabscience), goat Anti-Rabbit IgG antibody (H +  L),peroxidase (1:10000, E-AB-1003, Elabscience). Finally, the bands were visualized using the enhanced chemiluminescence (ECL) reagents (BL523A, Biosharp) and were quantified with ImageJ software.

### 2.15. Transmission electron microscopy (TEM)

The cortical tissue blocks fixed with 4% glutaraldehyde were taken out and then fixed in 1% osmotic acid for 30 min. After dehydration, the sections were embedded with resin and stained with uranyl acetate and lead citrate at 50 nm. The cortical microvascular structure and astrocyte ultrastructure were observed under transmission electron microscope.

### 2.16. Data statistics

SPSS 20.0 statistical software was used for statistical analysis, which was expressed in the form of mean ±  standard deviation (‾x ±  s). Two-way ANOVA with Bonferroni correction was used. P value <  0.05 was considered statistically significant. The histogram was made using Graph Pad Prism 8.4.0 (San Diego, California, United States).

## 3. Results

### 3.1. Aging and blood pressure measurement in WKY and SHRs

**Fig 1:**
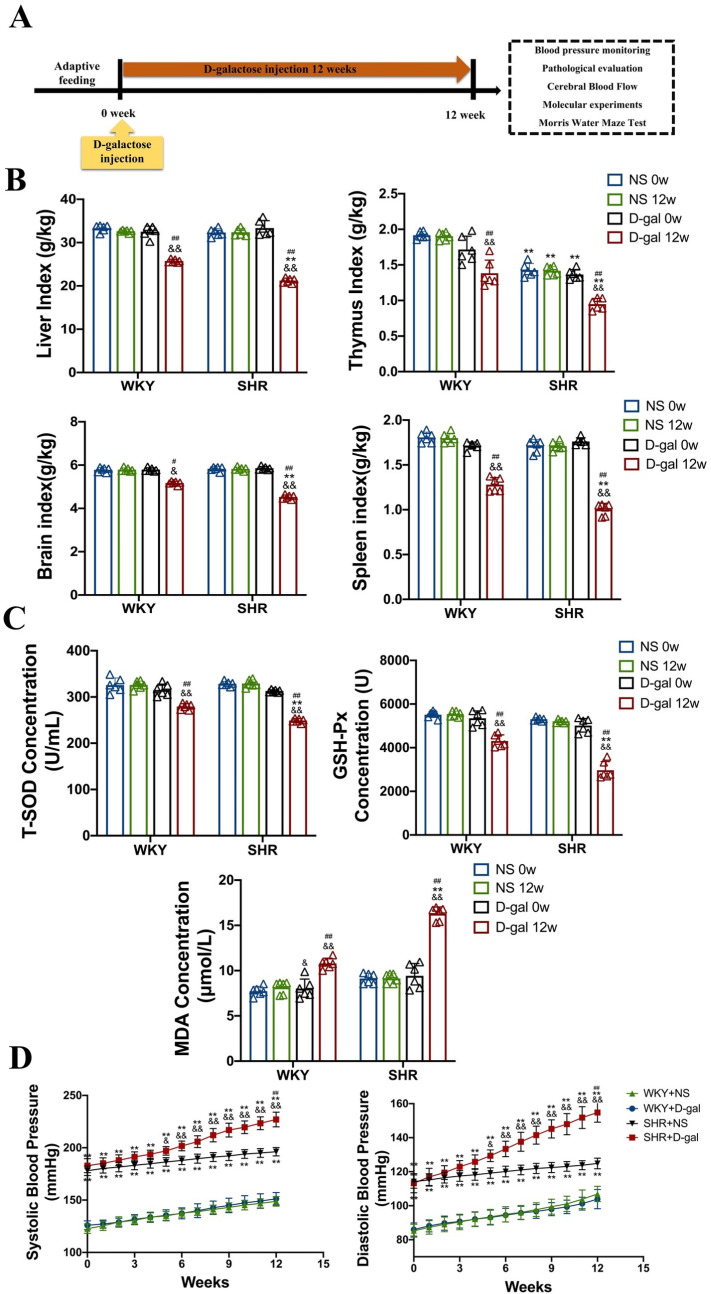
Changes in markers of aging and blood pressure in WKY and SHRs. (A) Animal handling flow chart. (B) Ratio of weight to body weight of liver, thymus, brain, and spleen at the time of rat sampling (N = 6). (C) Levels of oxidative stress in the serum of rat, including T-SOD, GSH-Px, and MDA (N = 6). (D) Weekly systolic and diastolic blood pressure changes in rats (N = 6). Data are presented as mean ±  SEM. The mean expression is shown as a relative expression level. Two-way ANOVA test:^&^vs NS; * vs WKY; ^#^vs D-gal 0w (^&^p < 0.05,^&&^p < 0.01, * p < 0.05, **p < 0.01,^#^p < 0.05, ^##^p < 0.01), respectively.

Hypertension persistently disrupts the structural and functional integrity of the cerebral microvascular system, compromises the integrity of the BBB, and promotes neuroinflammation and cognitive decline [14]. However, it remains unclear whether aging aggravates those above effects in SHRs. Therefore, we used SHRs with similar stages of hypertensive development to humans and injected SHRs with D-gal to induce aging (Fig 1A). As expected, the markers of liver, thymus, brain, and spleen were significantly reduced by 35%, 33%, 30%, 42% in the SHR + D-gal 12w group compared to the SHR + D-gal 0w group (P < 0.01, Fig 1B), and similar results were obtained in WKY. As shown in Fig 1C, the activity of T-SOD and GSH-Px in the serum of SHRs injected with D-gal for 12 weeks was immensely reduced by 24% and 55% (P < 0.05, P < 0.01), and the MDA content was extensively increased by 41% (P < 0.01). The above results suggest that D-gal can accelerate senescence in SHRs. To further examine the effects of senescence on SHRs, we monitor blood pressure weekly. It was found that both systolic and diastolic blood pressure were remarkablely higher in SHRs compared to WKY, with or without D-gal injection (P < 0.01,Fig 1D).

### 3.2. Aging exacerbates cognitive dysfunction caused by hypertension

**Fig 2:**
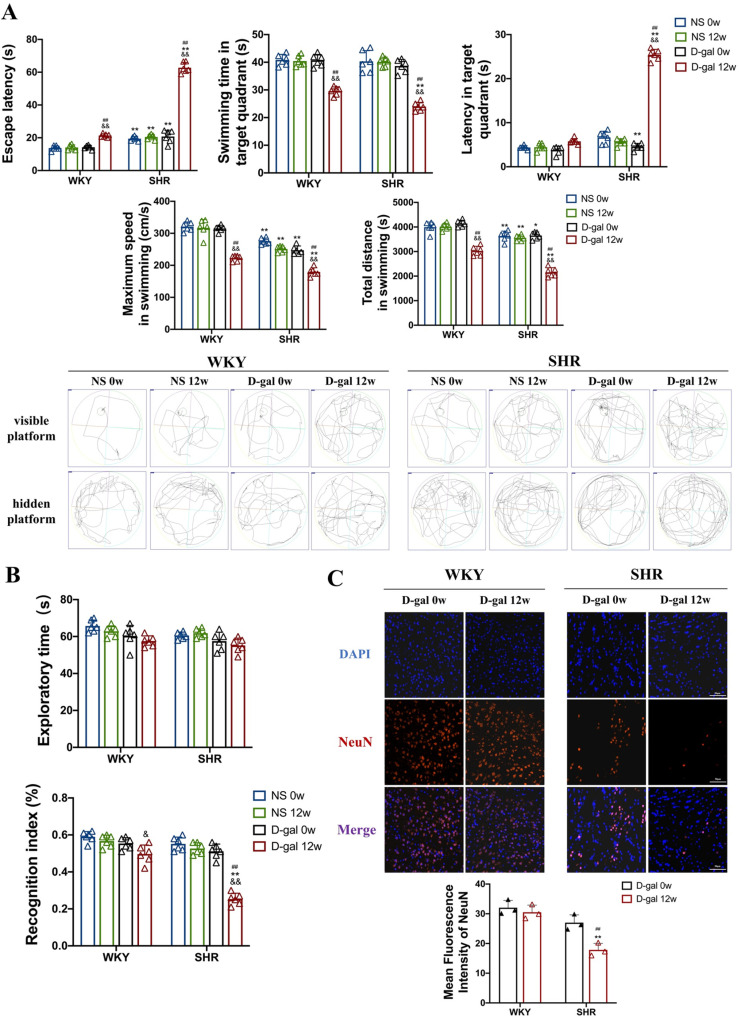
Cognitive impairment in WKY and SHRs. (A) The escape latency, swimming time in the target quadrant, latency in the target quadrant, maximum swimming speed and total swimming distance shown in MWM, and the navigation trials (up: navigation trial; down: probe trial)(N = 6). (B) The exploratory time and recognition index in NOR test (N = 6). (C) IF shows NeuN^ + ^-tagged neurons in the cortex of rat (40×)(N = 3). Data are presented as mean ±  SEM.The mean expression is shown as a relative expression level. Two-way ANOVA test: ^&^vs NS; * vs WKY; ^#^vs D-gal 0w (^&^p < 0.05, ^&&^p < 0.01, * p < 0.05, **p < 0.01,^#^p < 0.05, ^##^p < 0.01), respectively.

Hypertension is a major cause of age-related cognitive impairment in human, possibly due to hypertension by disrupting neurovascular coupling (NVU)[15]. However, whether aging exacerbates cognitive dysfunction in SHRs remains unclear. To determine the relationship between cognitive dysfunction and hypertension and aging, we first used MWM experiments to analyze cognitive function. The results showed that compared with WKY, SHRs had significant cognitive dysfunction, which was manifested by an extended escape latency, an increase latency in the target quadrant, a shortened swimming time in target quadrant, a decrease in maximum swimming speed, and a shortening of the total swimming distance. Apparently, this impairment was exacerbated in SHRs injected with D-gal for 12 weeks (P < 0.05, P < 0.01, Fig 2A).

NOR test is a behavioral experiment widely used to assess memory and cognitive abilities in animals. It was found that the recognition index of SHRs injected with D-gal for 12 weeks was vastly reduced by 59% compared to SHRs injected with D-gal for 0 week (P < 0.05, P < 0.01, Fig 2B). The results proved that the memory ability of SHRs induced by D-gal was enormously weakened.

Additionally, aberrant neuronal activity and damage have been found to be associated with cognitive dysfunction [16], so we used NeuN^ +^ to label neurons. The results showed that the mean fluorescence intensity of NeuN^ +^ markers in the cortex of SHRs injected with D-gal for 12 weeks was vastly reduced by 44% and 36% compared with that of WKY and SHRs injected with D-gal for 0 week, confirming the loss of cortical neurons in senescent SHRs (P < 0.01, Fig 2C). Therefore, these results demonstrate that D-gal-induced senescence may exacerbate memory impairment and congnitive dysfunction in SHRs.

### 3.3 Cerebral microvascular injury occurred in elderly hypertensive rats

**Fig. 3.**
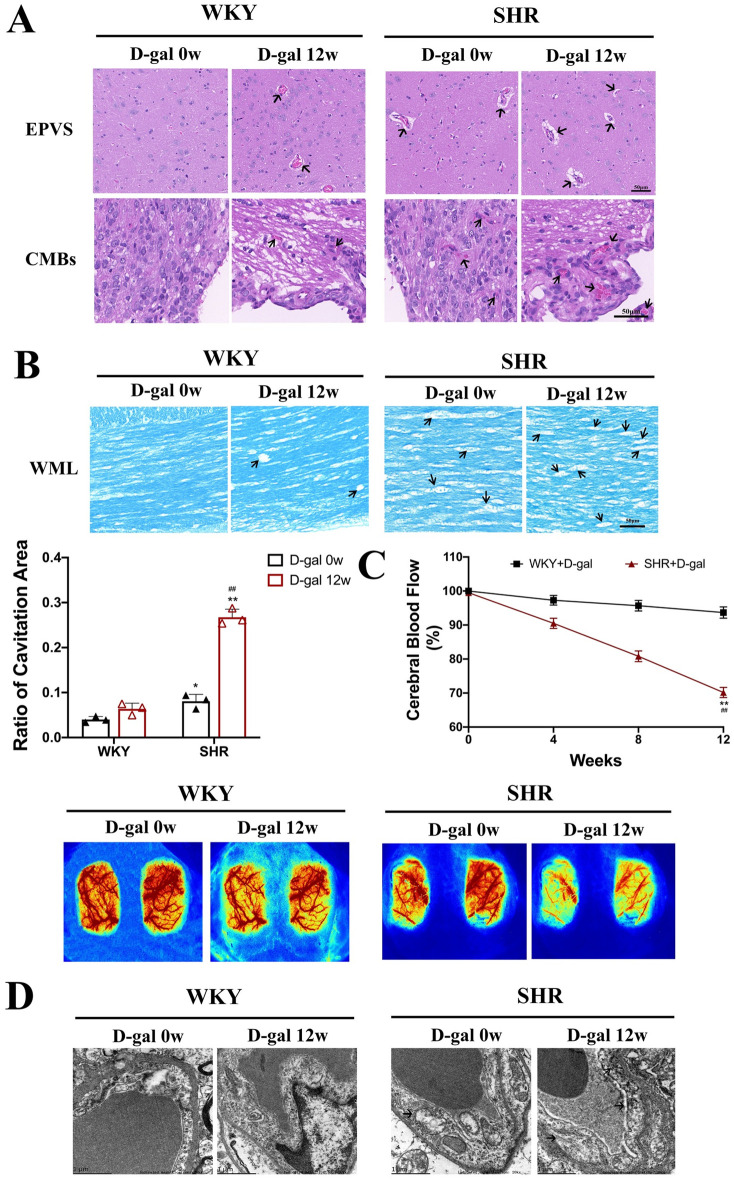
Cerebral microvascular injury in WKY and SHRs. (A) HE staining showed EPVS (40×) and CMBs in the dorsal third ventricle (40×)(N = 3). (B) LFB staining showed the proportion of WML and myelin vacuole area of corpus callosum in rats (40×)(N = 3). (C) Changes in CBF at 0 and 12 weeks for the same WKY and SHRs (N = 6). (D) TEM showing microvascular changes in the deep cortical region of rats(N = 3). Data are presented as mean ±  SEM.The mean expression is shown as a relative expression level. Two-way ANOVA test: * vs WKY; ^#^vs D-gal 0w (*p < 0.05, **p < 0.01, ^#^p < 0.05, ^##^p < 0.01), respectively.

Previous MRI results and some clinical studies[17–19] have found that when the integrity of cerebral microvessels is impaired, enlarged perivascular space (EPVS) and cerebral microbleeds (CMBs) occur in older adults and hypertensive patients. However, it is unclear whether aging exacerbates cerebral microvascular injury in SHRs. To determine the relationship between cerebral microvascular injury and hypertension and aging, we first performed HE staining of rat brain tissue. The results showed that WKY and SHRs injected with D-gal for 12 weeks had a wider range of EPVS and CMBs compared to WKY injected with D-gal for 0 week (Fig 3A). In addition, we performed LFB staining on brain tissue of SHRs to visualize white matter lesions (WML). As shown in Fig 3B, the myelin vacuolar area of the corpus callosum was obviously increased by 65% in SHRs injected with D-gal for 12 weeks compared to SHRs injected with D-gal for 0 week (P < 0.05, P < 0.01).

On this basis, we also evaluated the structure and function of cerebral microvessels in SHRs. CBF is a crucial support for cerebral microcirculation, so we measured CBF. The results showed a 22% decrease in CBF in rats in the SHR + D-gal 12w group compared to the WKY + D-gal 12w group (P < 0.01, Fig 3C). Finally, we also used TEM to detect cortical microvascular changes and found that SHRs injected with D-gal for 12 weeks had more significant cortical microvascular swelling, reduced mitochondrial crests, and endoplasmic reticulum folding than SHRs injected with D-gal for 0 week (Fig 3D). These results declare that D-gal-triggered senescence can induce more severe pathological changes and structural and functional impairment of cerebral microvessels in SHRs.

### 3.4 Aging exacerbates the disruption of the BBB and neuroinflammation caused by hypertension

**Fig 4:**
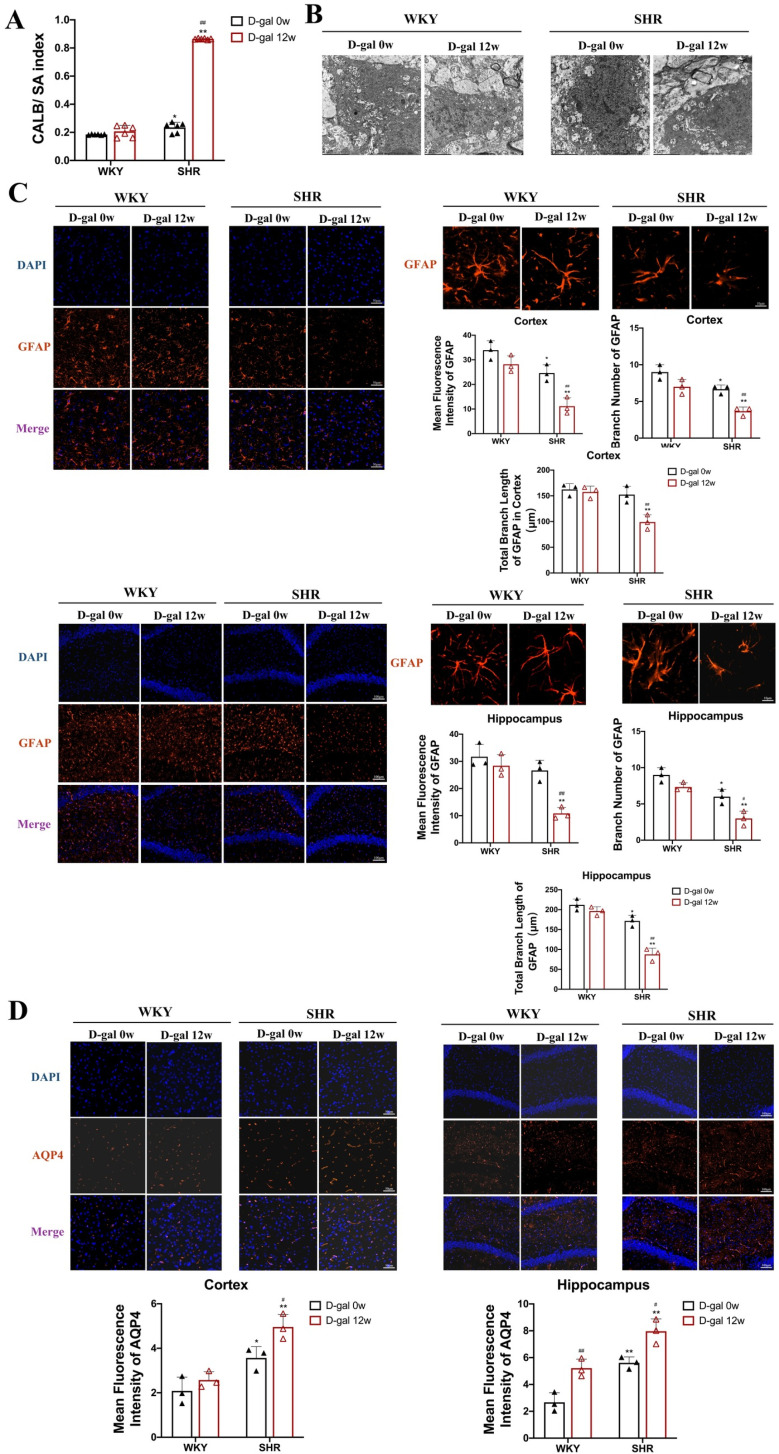
Permeability of the BBB in WKY and SHRs. (A) Ratio of CALB/SA in the serum of rats (N = 6). (B) TEM showing the ultrastructure of astrocytes in the deep cortex of rats(N = 3). (C) Expression levels in the cortex and hippocampal astrocytes of rats (40×) and representative images of astrocytes immunostaining GFAP, branch number, and total branch length(200×)(N = 3). (D) Expression of AQP4 in the cortex and hippocampus astrocytes of rats (40×)(N = 3). Data are presented as mean ±  SEM.The mean expression is shown as a relative expression level(N = 6). Two-way ANOVA test: * vs WKY; ^#^vs D-gal 0w (*p < 0.05, **p < 0.01, ^#^p < 0.05,^##^p < 0.01), respectively.

Previous studies have confirmed that BBB dysfunction is a common occurrence in hypertension and the elderly[20], and the ratio of cerebrospinal fluid to serum albumin (CALB/SA) is one of the classic indicators for the detection of BBB, so we first analyzed the cerebrospinal fluid of SHRs. The results showed that, as shown in Fig 4A, the CALB/SA ratio of SHRs injected with D-gal for 12 weeks was significantly increased by 76% compared to WKY and SHRs injected with D-gal for 0 week (P < 0.05,P < 0.01). Since astrocyte is one of the important components of the BBB[21], the function and morphology of astrocyte was also tested. TEM results show extensive astrocyte edema in SHRs injected with D-gal for 12 weeks compared to WKY and SHRs injected with D-gal for 0 week (Fig 4B).

On this basis, we also examined the histological changes in astrocytes of SHRs. The results of GFAP immunofluorescence showed (Fig 4C) that the expression of GFAP in the cortex of SHRs was obviously down-regulated by 69% and 64%, respectively, and by 70% and 63% in the hippocampus compared with WKY and SHRs injected with D-gal for 0 week (P < 0.01). The number of astrocyte branches in the cortex of SHRs decreased by 56% and 43% and decreased by 67% and 57% in the hippocampus (P < 0.05, P < 0.01). The total length of astrocyte branches in the cortex of SHRs decreased by 44% and 40%, respectively, and decreased by 62% and 50% in the hippocampus (P < 0.01).

In terms of astrocyte function, AQP4 is involved in maintaining the integrity of BBB as an aquaporin[22]. AQP4 expression has been found to increase in SHRs [23] and significantly with aging[24]. The results showed that, as shown in Fig 4D, the positive expression of AQP4 in the cortex of rats in the SHR + D-gal 12w group increased by 54% and 24% compared with the WKY group and SHR + D-gal 0w group (P < 0.05, P < 0.01), and increased by 37% and 30% in the hippocampus, respectively (P < 0.05, P < 0.01).

**Fig 5:**
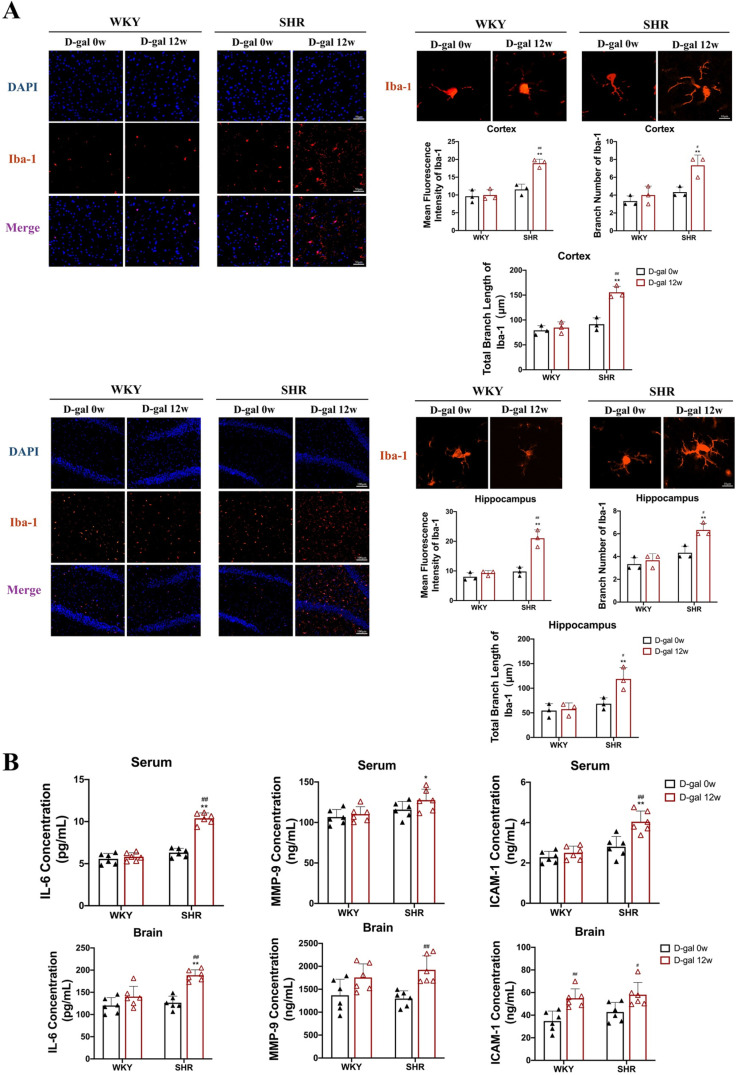
Activation and inflammation levels of microglia in WKY and SHRs. (A) Expression levels in the cortex and hippocampal microglia of rats (40×) and representative images of microglial immunostaining Iba-1, branch number, and total branch length(200×)(N = 3). (B) Levels of inflammatory markers, including IL-6, MMP-9, and ICAM-1, in the serum and brain tissue of rats (N = 6). Data are presented as mean ±  SEM.The mean expression is shown as a relative expression level. Two-way ANOVA test: * vs WKY; ^#^vs D-gal 0w (*p < 0.05, **p < 0.01,^#^p < 0.05, ^##^p < 0.01), respectively.

It is well-known that hypertension not only impairs BBB integrity, but also induces neuroinflammation[25]. To investigate alterations in neuroinflammation, we further assessed microglial activity in the cortex and hippocampus of rats. As shown in Fig 5A, the expression levels of Iba-1 in the cortex and hippocampus of SHRs injected with D-gal for 12 weeks were up-regulated by 33% and 50%, respectively compared with SHRs injected with D-gal for 0 week (P < 0.01). At the same time, the number of microglial branches in the cortex and hippocampus was widely increased by 43% and 33% (P < 0.05, P < 0.01), and the total branch length decreased by 40% and 50% (P < 0.05, P < 0.01), indicating that most microglia were activated and inflammation was intensified. Meanwhile, we also detected neuroinflammatory markers MMP-9, ICAM-1, and IL-6 in serum and brain. As shown in Fig 5B, the levels of IL-6 in serum and brain tissue, and MMP-9 levels in brain tissue increased by 40%, 33%, and 23% in the SHR + D-gal 12w group compared to the SHR + D-gal 0w group (P < 0.01). In addition, ICAM-1 in serum was also increased by 43% in SHRs injected with D-gal for 12 weeks compared with SHRs injected with D-gal for 0 week (P < 0.01). These results indicate that aging exacerbates hypertension-mediated BBB damage and promotes microglial activation and neuroinflammation.

### 3.5 Aging contributes to cerebral microvascular damage via endothelial dysfunction

**Fig 6:**
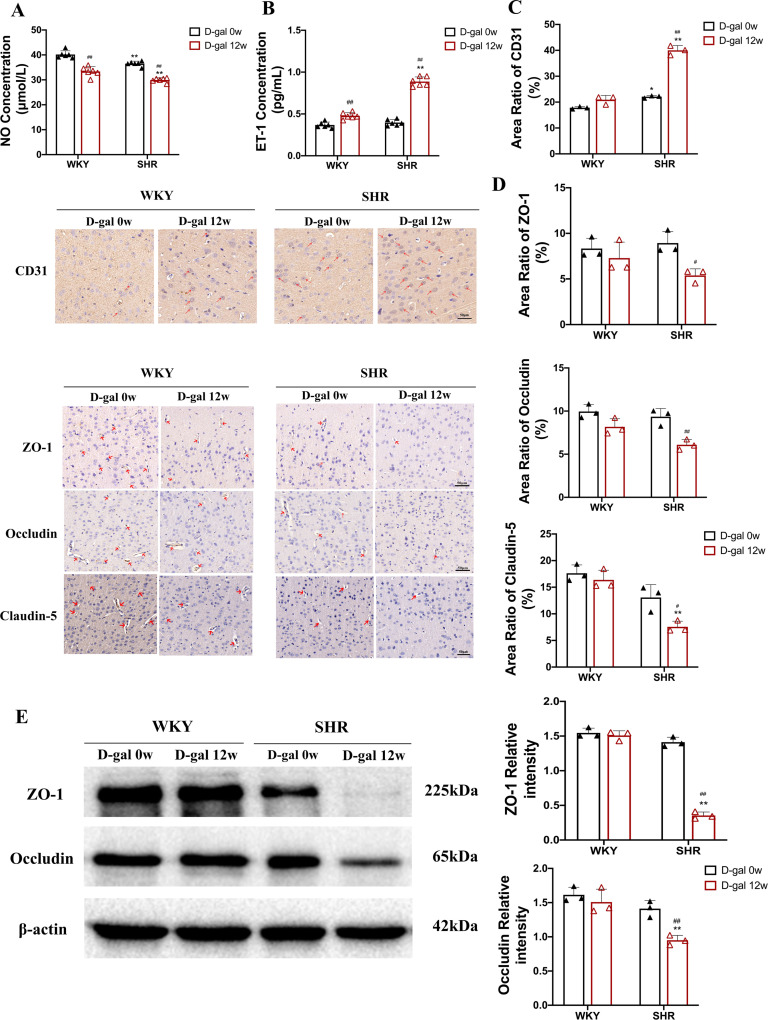
Cerebral microvascular endothelial dysfunction in WKY and SHRs. (A-B) Indicators showing vasodilator and systolic function, including NO and ET-1(N = 6). (C) Immunohistochemistry showed changes in microvascular density of CD31^ +^ markers in the cortex of rats (40×)(N = 3). (D) Immunohistochemistry showed the ratio of the expression area of tight junction proteins in the cortex of rats such as ZO-1, Occludin, and Claudin-5 (40×)(N = 3). (E) WB shows relative expression of endothelial tight junction proteins, including ZO-1, Occludin, in the rat cortex (N = 3). Data are presented as mean ±  SEM. The mean expression is shown as a relative expression level. Two-way ANOVA test: * vs WKY;^#^vs D-gal 0w (*p < 0.05,**p < 0.01,^#^p < 0.05, ^##^p < 0.01), respectively.

Recent studies have demonstrated that age-related endothelial dysfunction is associated with impaired angiogenesis processes and subsequent pathological remodeling of the microcirculation, which results from a decrease in NO due to oxidative stress in the endothelium[26,27]. Therefore, we first measure the level of NO in the serum of SHRs. The results of Fig 6A show that SHRs injected with D-gal for 12 weeks have 18% and 20% reduction in NO levels compared to WKY and SHRs injected with D-gal for 0 week (P < 0.01). ET-1 has been reported to be the strongest vasoconstrictor peptide produced by vascular endothelial cells in *vivo*, which is present in many diseases related to endothelial dysfunction and plays an important role in regulating vascular homeostasis [28]. The results showed a 38% and 50% increase in ET-1 levels in SHRs injected with D-gal for 12 weeks compared to WKY and SHRs injected with D-gal for 0 week (P < 0.01, Fig 6B).

Moreover, CD31^ +^ is a vascular endothelial cell marker that has been shown to play a vital role in ECs migration and angiogenesis, which is closely related to aging [29]. It has been found that hypertensive rats exhibit higher circulating CD31^ +^ [30]. We found that the expression of CD31^ +^ in the cortical region of SHRs injected with D-gal for 12 weeks was significantly increased by 48% compared to SHRs injected with D-gal for 0 week (P < 0.01, Fig 6C).

It has also been found that tight junction proteins are proteins that regulate the permeability of the epithelial and endothelial tissue barriers, including ZO-1, Occludin, Claudin-5 and are closely related to endothelial dysfunction [31,32]. Therefore, WB and immunohistochemistry were used to detect the expression of endothelial tight junction protein in the cortex of rats. The results of immunohistochemistry showed that the protein expressions of ZO-1, occludin and claudin-5 in the cortex of SHRs injected with D-gal for 12 weeks decreased by 38%, 32% and 37%, respectively compared with SHRs injected with D-gal for 0 week (P < 0.05, Fig 6D). Meanwhile, the WB results showed that the expression of ZO-1 and occludin in the cortex of SHRs injected with D-gal for 12 weeks decreased by 72% and 43%, respectively compared with SHRs injected with D-gal for 0 week (P < 0.01, Fig 6E). These results illustrate that aging leads to cerebral microvascular injury by influencing cerebral microvascular constriction, increasing damaged endothelial synthesis and release, producing vascular endothelial differentiation and reducing endothelial cell tight junctions.

### 3.6 Combination of nimodipine and butylphthalide in the treatment of cognitive dysfunction in senescence-induced SHRs

**Fig 7:**
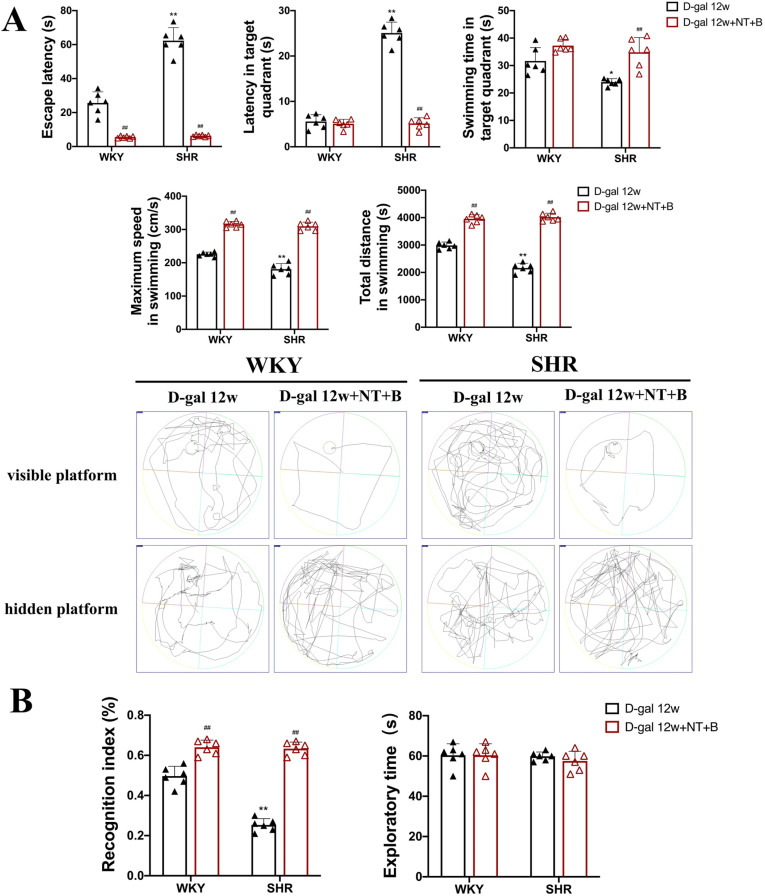
Treatment of cognitive dysfunction in WKY and SHRs. (A) The escape latency, latency in the target quadrant, swimming time in the target quadrant, maximum swimming speed and total swimming distance in MWM, and the navigation trials (up: navigation trial; down: probe trial)(N = 6). (B) The exploratory time and recognition index in NOR test (N = 6). Data are presented as mean ±  SEM.The mean expression is shown as a relative expression level. Two-way ANOVA test: * vs WKY; ^#^vs D-gal 12w (*p < 0.05,**p < 0.01,^#^p < 0.05, ^##^p < 0.01), respectively.

The previous findings of our research group have found that D-gal-induced senescence combined with cerebral hypoperfusion can lead to cognitive dysfunction in SHRs [33]. Therefore, we used nimodipine [12] for the clinical treatment of hypertension and butylphthalide [34] for the treatment of cerebral ischemia in combination for the treatment of cognitive dysfunction in WKY and SHRs. As shown in Fig 7A, the escape latency and latency in the target quadrant of SHRs treated with nimodipine and butylphthalide were importantly reduced by 83% and 80% compared with the WKY/SHR + D-gal 12w group (P < 0.01). More importantly, the combination of nimodipine and butylphthalide also immensely increased the swimming time in the target quadrant by 29%, the maximum swimming speed by 40%, and the total swimming distance by 50% in SHRs (P < 0.01).

As shown in Fig 7B, the combination of nimodipine and butylphthalide increased the recognition index of WKY and SHRs injected with D-gal for 12 weeks by 26% and 54% (P < 0.01), but did not change the exploration time of WKY and SHRs. In summary, the above results reveal that nimodipine and butylphthalide can be combined in the treatment of cognitive dysfunction in aging-induced SHRs.

## 4. Discussion

The main findings of this study: (1) EC in cerebral microvessels is the main target of aging, and its pathogenesis is mainly manifested as abnormal NO synthesis, ultimately leading to endothelial dysfunction; (2) Aging-induced endothelial dysfunction, combined with cerebral microvascular injury, BBB injury, and neuroinflammation, exacerbates cognitive dysfunction (learning and spatial memory loss) in SHRs; (3) Classical clinical treatment of hypertension and intracerebral hypoperfusion: the combination of nimodipine and butylphthalide can effectively improve the cognitive dysfunction of SHRs. Therefore, these data provide strong evidence for the mechanistic link between aging and hypertension and cognitive decline, and strengthen the biological hypothesis that aging-induced endothelial dysfunction is undertaken.

Clinical studies have found that hypertension and aging are risk factors for cognitive impairment [35], and age is also a pivotal risk factor for cognitive dysfunction in patients with hypertensive. Consistent with clinical results, chronic hypertension has also been found in animal experiments to induce cerebrovascular disease, accelerating age-related cognitive decline [36, 37]. Here, we demonstrate that cognitive impairment in SHRs aggravated by aging is associated with cerebral microvascular injury. Accumulating evidence suggests that cerebrovascular dysfunction precedes the development of cognitive dysfunction [38]. Interestingly, cerebral microvessels maintain healthy cognitive function by relying on vasodilation (functional congestion) of neuronal activity for transient adjustments in CBF [39]. In recent clinical studies, it is not difficult to find that white matter hyperintensity(WMH), CMBs, and EPVS are pathological markers associated with cerebral small vessel disease (CSVD), and their appearance is also a marker of cerebral microvascular injury [40]. In our report, the above pathological changes, including WML, CMBs, EPVS, were also found in SHRs, and the severity of these events was exacerbated by 12-week senescence induced by D-gal injection. This is consistent with several previous studies [41, 42]. To support this conclusion, we further targeted treatment for vascular dysfunction followed by cognitive assessment. Nimodipine has been found to protect vascular function and cognitive function in animal models of cerebral small vessel disease[43] and improve vascular endothelial function in hypertensive patients [44]. Another study found that butylphthalide could improve cognitive function and vascular endothelial function in patients with subarachnoid hemorrhage complicated by cerebral vasospasm [45]. Therefore, in our study, nimodipine and butylphthalide were used in combination to treat cognitive dysfunction in D-gal aggravated SHRs. The results showed that the combination of the two drugs could effectively reduce the escape latency and target quadrant latency, increase the swimming time, maximum swimming speed and total swimming distance in the target quadrant, and effectively improve the cognitive index of SHRs, confirming that these two drugs could improve the cognitive impairment of SHRs.

Clinical studies have linked endothelial dysfunction to cognitive decline in elderly cardiovascular and cerebrovascular patients [46, 47], but the exact mechanism of this association is unclear. Therefore, we hypothesized that senescence mediates abnormal brain microvascular NO synthesis, resulting in endothelial dysfunction and ultimately aggravating cognitive impairment in SHRs. Previous studies have reported that aging and hypertension independently impair endothelial dysfunction, and this effect may be due to a decrease in NO [48]. In this study, it was found that SHRs injected with D-gal for 12 weeks had reduced NO content in serum under the dual stimulation of hypertension and aging, which is consistent with the results of previous in *vitro* and in *vivo* studies [49, 50]. Nevertheless, this study has not yet examined whether senescence reduces NO bioavailability and eNOS activity and its conjugates in SHRs, which warrants further investigation. Moreover, ET-1-dependent contractile of blood vessels has been found to increase in hypertensive patients, and ET-1 activity increases with age [51]. Similar results were obtained in this study, with significantly higher ET-1 levels in SHRs injected with D-gal for 12 weeks than in WKY and SHRs injected with D-gal for 0 week. CD31^ +^ is known to be the most abundant constitutive co-signaling receptor glycoprotein on endothelial cells and plays a crucial role in maintaining vascular interface homeostasis, cell migration, and angiogenesis [52]. Previous studies have found that carvacrol, a monomer of traditional Chinese medicine, can improve vascular function in hypertensive animals by decreasing vascular ROS, increasing CD31^ +^ expression, improving endothelial repair induced by endothelial progenitor cells (EPCs), and alleviating endothelial dysfunction [53]. In this study, immunohistochemical detection showed that the expression of CD31^ +^ was increased, suggesting that the cerebral microvascular density of elderly SHRs injected with D-gal for 12 weeks was reduced. Consequently, future studies need to elucidate the specific mechanism of the effect of cerebral microvascular density reduction on cognitive function impairment in elderly hypertensive rats. Tight junction proteins are well-known proteins that regulate the permeability of the epithelial and endothelial tissue barriers [54]. Accordingly, this study also examined tight junction proteins that are closely associated with endothelial dysfunction, including ZO-1, Occludin, and Claudin-5, and found that tight junction protein expression was essentially reduced in the SHRs injected with D-gal for 12 weeks. This is consistent with the results of several previous studies [55, 56]. These results suggest that senescence aggravates endothelial dysfunction in SHRs, thereby aggravating cognitive dysfunction. Hence, we hypothesize that cognitive dysfunction due to endothelial dysfunction may be related to a decrease in NO, an increase in ET-1 and CD31^ + ^, and a decrease in tight junction proteins.

However, the causes of cognitive dysfunction exacerbated by aging in SHRs may be multifactorial. For example, the neurovascular unit, known as the BBB, formed by cerebral microvascular endothelial cells surrounded by pericytes and astrocytes, is often associated with cognitive decline in animals and humans [57]. Therefore, we suspect that aging may lead to irreversible cerebrovascular damage, destruction of neurovascular units, and thus aggravation of cognitive impairment in SHRs. Previous studies have also reported that AngII exacerbates BBB damage in elderly hypertensive mice, and it is well known that BBB damage increases the CALB/SA ratio [58]. In our experiments, we also found a substantial increase in the CALB/SA ratio in SHRs injected with D-gal for 12 weeks compared to SHRs injected with D-gal for 0 week. Interestingly, we also found that GFAP expression was decreased in the cerebral cortex and hippocampus of senescence-induced SHRs injected with D-gal for 12 weeks, and positive expression of AQP4 was increased. These data are consistent with those found in SHRs and the elderly [59,60]. It is well known that neuroinflammation plays an all-important role in the process of hypertension, as does BBB injury [61]. In our results, we found that MMP-9, ICAM-1, and IL-6 levels were elevated in serum and brain tissue of SHRs, and microglia were activated, while these indicators changed more significantly 12 weeks after D-gal injection. These results suggest that aging exacerbates neuroinflammation during hypertension, thereby exacerbating cognitive dysfunction in SHRs.

## 5. Conclusion

The results of this study provide an epoch-making theoretical basis for the hypothesis that endothelial dysfunction aggravates cognitive dysfunction in SHRs, and further recognizes that impaired NO synthesis during aging is a key cause of endothelial dysfunction. This may lead to age-specific treatments for cognitive dysfunction.

## Supporting information

S1 File WB raw images. (PDF)

S2 File raw data. (XLSX)
