# Supplementary material for: Aging aggravates cognitive dysfunction in spontaneously hypertensive rats by inducing cerebral microvascular endothelial dysfunction
Source: PLoS One. 2025 Mar 13;20(3):e0316383. doi: 10.1371/journal.pone.0316383 (PMC11906062; doi:10.1371/journal.pone.0316383)
Supplement: S1 File — (PDF) [file pone.0316383.s001.pdf]

ZO-1 225kDa (6%SDS-PAGE)

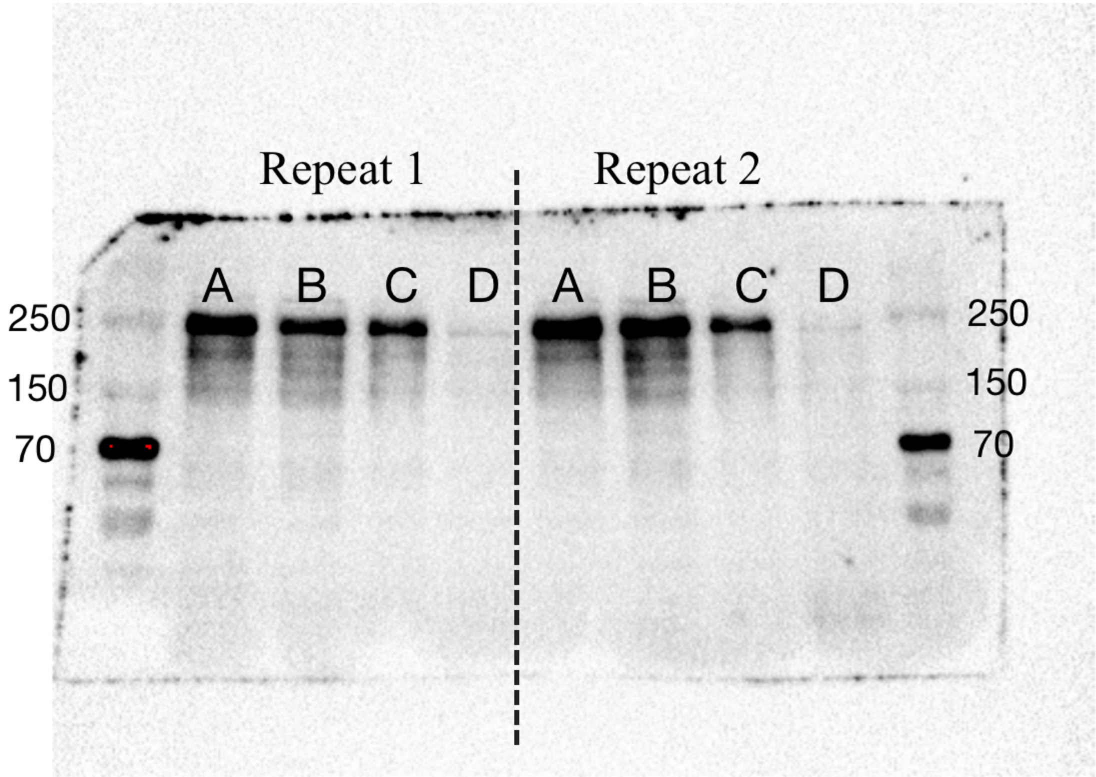

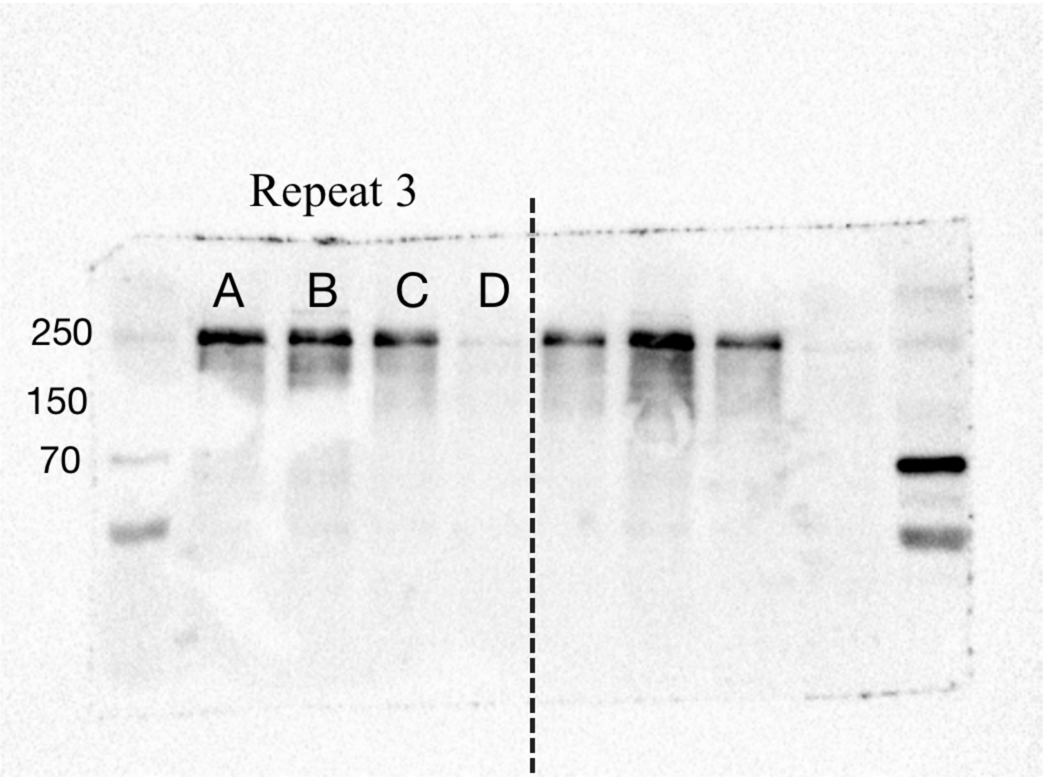

The raw data of Western blotting experiment.

A:WKY+D-gal 0w (young)

B:WKY+D-gal 12w (old)

C:SHR+D-gal 0w (young)

D:SHR+D-gal 12w (old)

Occludin 65kDa (10%SDS-PAGE)

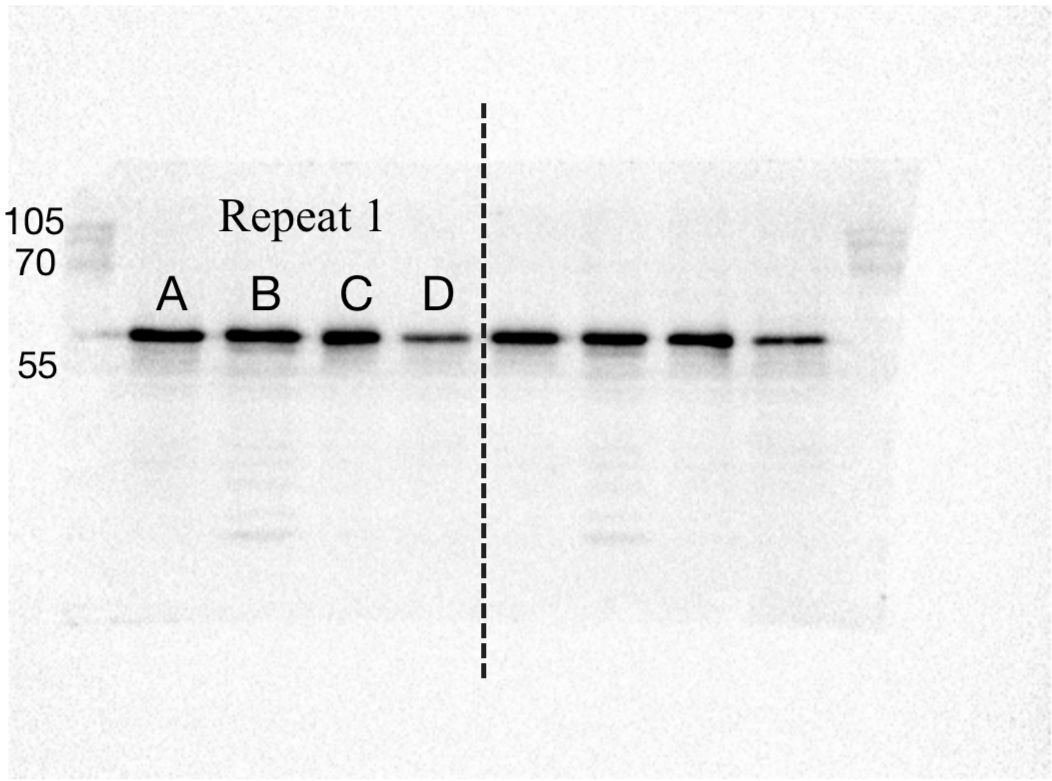

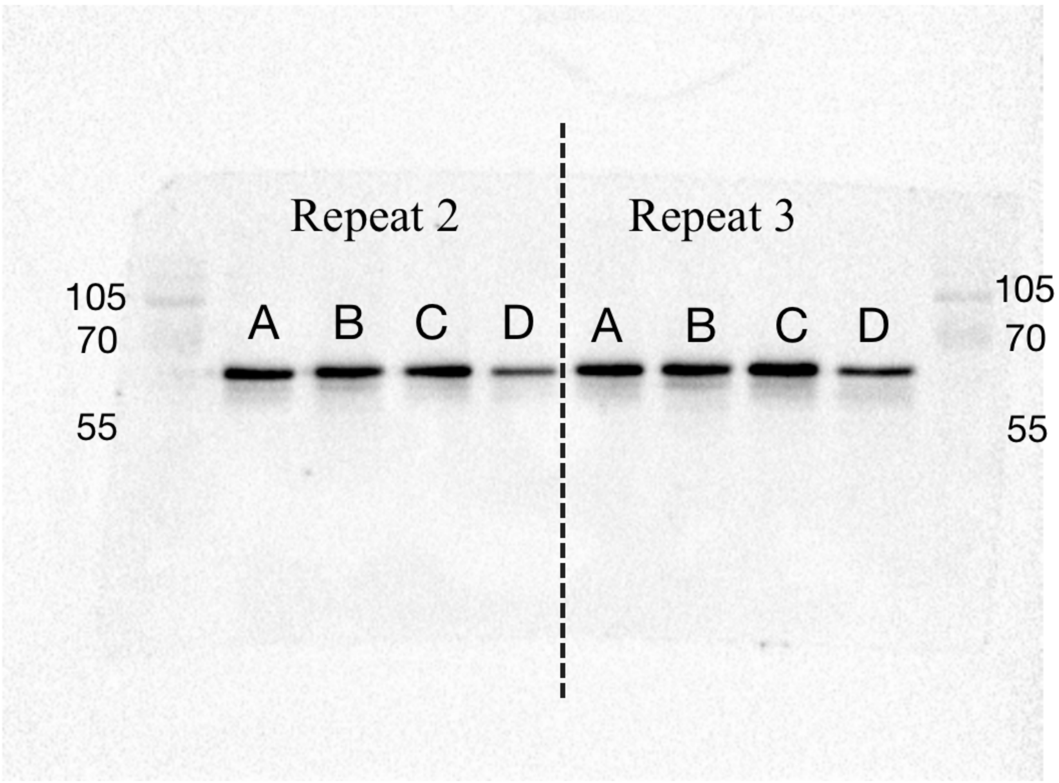

The raw data of Western blotting experiment.

A:WKY+D-gal 0w (young)

B:WKY+D-gal 12w (old)

C:SHR+D-gal 0w (young)

D:SHR+D-gal 12w (old)

β-actin 42kDa （10%SDS-PAGE)

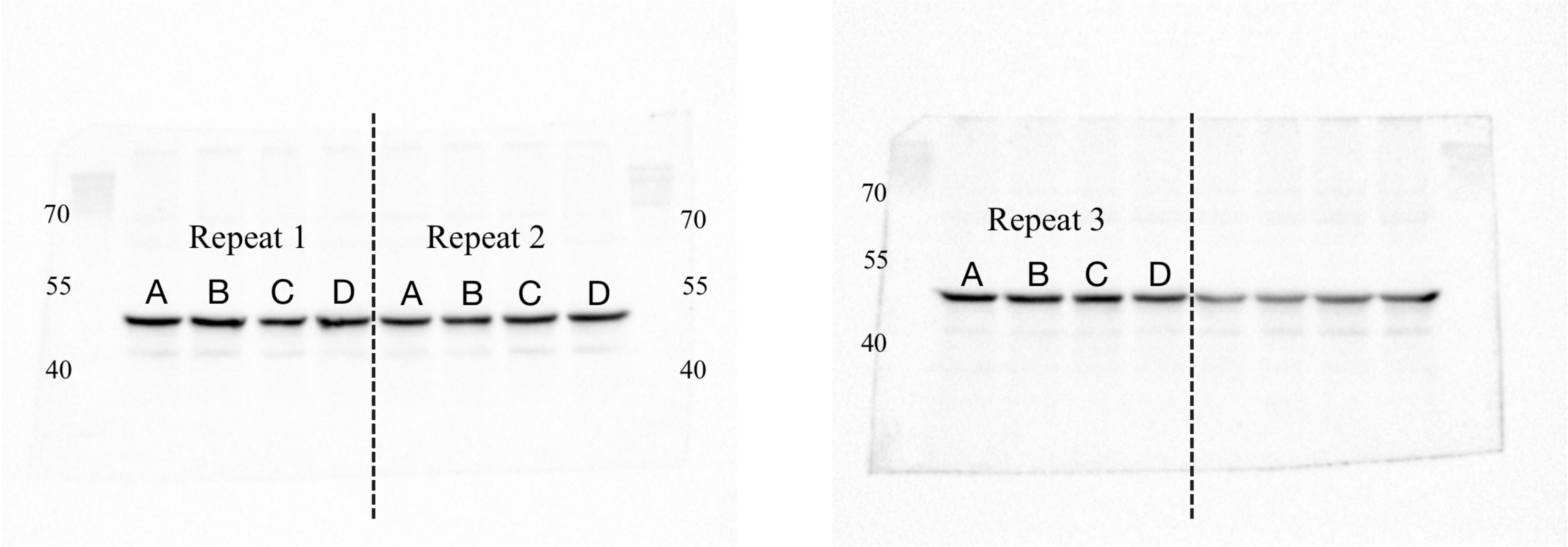

The raw data of Western blotting experiment.

A:WKY+D-gal 0w (young)

B:WKY+D-gal 12w (old)

C:SHR+D-gal 0w (young)

D:SHR+D-gal 12w (old)
